# Targeting Cdc42 with the small molecule drug AZA197 suppresses primary colon cancer growth and prolongs survival in a preclinical mouse xenograft model by downregulation of PAK1 activity

**DOI:** 10.1186/1479-5876-11-295

**Published:** 2013-11-27

**Authors:** Karin Zins, Sandun Gunawardhana, Trevor Lucas, Dietmar Abraham, Seyedhossein Aharinejad

**Affiliations:** 1 Laboratory for Molecular Cellular Biology, Center for Anatomy and Cell Biology, Medical University of Vienna, Waehringerstrasse 13, A-1090 Vienna, Austria

**Keywords:** Colon cancer, Small molecule inhibitor, Rho GTPases, Cdc42, Cancer therapy, Xenograft model

## Abstract

**Background:**

Rho GTPases play important roles in cytoskeleton organization, cell cycle progression and are key regulators of tumor progression. Strategies to modulate increased Rho GTPase activities during cancer progression could have therapeutic potential.

**Methods:**

We report here the characterization of a Cdc42-selective small-molecule inhibitor AZA197 for the treatment of colon cancer that was developed based on structural information known from previously developed compounds affecting Rho GTPase activation. We investigated the effects of AZA197 treatment on RhoA, Rac1 and Cdc42 activities and associated molecular mechanisms in colon cancer cells *in vitro*. Therapeutic effects of AZA197 were examined *in vivo* using a xenograft mouse model of SW620 human colon cancer cells. After treatment, tumors were excised and processed for Ki-67 staining, TUNEL assays and Western blotting to evaluate proliferative and apoptotic effects induced by AZA197.

**Results:**

In SW620 and HT-29 human colon cancer cells, AZA197 demonstrated selectivity for Cdc42 without inhibition of Rac1 or RhoA GTPases from the same family. AZA197 suppressed colon cancer cell proliferation, cell migration and invasion and increased apoptosis associated with down-regulation of the PAK1 and ERK signaling pathways *in vitro*. Furthermore, systemic AZA197 treatment reduced tumor growth *in vivo* and significantly increased mouse survival in SW620 tumor xenografts. Ki-67 staining and tissue TUNEL assays showed that both inhibition of cell proliferation and induction of apoptosis associated with reduced PAK/ERK activation contributed to the AZA197-induced therapeutic effects *in vivo*.

**Conclusions:**

These data indicate the therapeutic potential of the small-molecule inhibitor AZA197 based on targeting Cdc42 GTPase activity to modulate colorectal cancer growth.

## Introduction

In recent years, the focus of cancer drug development has shifted from conventional broad spectrum cytotoxic drugs to therapeutics specifically targeting the molecular mechanisms driving the development of cancer. The Rho family proteins Rac1, Cdc42 and RhoA are small GTP-binding proteins regulating multiple cellular processes such as cell cytoskeleton organization, cell cycle progression and cell migration [1]. Rho family members act as molecular switches, cycling between an inactive, GDP-bound form and an active, GTP-bound form that determine the cellular functions of Rho GTPases [2]. Rho GTPase activity is modulated by differential activation of Rho GTPase regulating signaling pathways and expression of Rho GTPase regulatory molecules such as guanine nucleotide exchange factors (GEFs) that increase Rho GTPase activity by promoting the release of bound GDP [1].

Unregulated Rho GTPase activity contributes to the development of proliferative malignancies such as colon carcinoma influencing proliferation, apoptosis, migration and invasion associated with cancer progression [3,4]. The discovery that Rho GTPases play important roles in tumor development and progression raised considerable interest in these proteins as potential targets for cancer therapy. A number of inhibitors either targeting Rho GTPase activity directly [1,5] or targeting regulators of Rho GTPase activity (e.g. GEFs) have been developed [2,6]. Although targeted drugs that inhibit Rho GTPases and downstream signaling kinases have not yet been widely adopted for clinical use, their potential value as cancer therapeutics continues to drive considerable pharmaceutical research and development [7].

Rac1 exerts tumor specific roles and is overexpressed in many tumors [3]. Much evidence support the importance of Rac1 in colorectal adenocarcinoma [8] and it has been shown that overexpression of Rac1 in colon cancer cells accelerates the tumorigenic process which may be suppressed by inhibition of Rac1 expression with RNA interference [9]. Increased RhoA expression has been described in various human tumors including colon cancer associated with malignant progression [10], although Rho GTPases also seem to have a tumor suppressive function since loss of Rho function is associated with predisposition to lymphoid cell transformation [11].

Cell division control protein 42 (Cdc42) is involved in cell cycle control and metastasis [12], and plays a role in the regulation of cell and migration polarity [13] inhibiting invasion by promoting epithelial polarity as well as stimulating migration [6]. Cdc42 expression is up-regulated in breast cancer [10], however loss of Cdc42 enhances liver cancer development [14], suggesting that the multiple roles of Cdc42 affect cancer progression in a tissue-specific manner. GTP-bound Cdc42 can interact with multiple downstream signaling pathways, including activation of p21-activated protein kinase (PAK), which is involved in invasion, migration and oncogenic transformation [15,16]. Additionally, PAK1 expression is significantly increased in colorectal cancer and closely correlates with aggressive disease progression [17]. Moreover, Cdc42 was found to be over-expressed with high incidence in colorectal cancer samples suggesting a potential role for Cdc42 in tumor development [18].

In this study, we identify a highly efficient small molecule anticancer agent AZA197 that specifically inhibits Cdc42. We report that, AZA197 reduces the proliferative potential of both HT-29 colorectal cancer cells and the highly invasive SW620 colorectal cell line associated with decreased PAK/ERK activation. Moreover, AZA197 decreases SW620 colon cancer cell migration and invasion. Studies *in vivo* showed that AZA197 reduces the growth of human SW620 colon cancer xenografts and significantly improves animal survival.

## Methods

### Cell lines and molecular profiling

3T3-Swiss fibroblasts (ATCC, Manassas, VA; CCL-92) and human SW620 (ATCC, CCL-227) and HT-29 (ATCC, HTB-38) colorectal adenocarcinoma cells were obtained from American Type Culture Collection (ATCC) and cultured in Dulbecco’s modified Eagle’s medium (DMEM, PAA Laboratories, Pasching, Austria) supplemented with 10% fetal calf serum (FCS; PAA Laboratories), 0.1 M nonessential amino acids (PAA Laboratories), 100 U/ml penicillin and 100 μg/ml streptomycin (culture medium). The SW620 cell line was tested for authenticity using STR-PCR (PowerPlex 16 HS System, Promega, Madison, WI).

### Compound generation

Based on the available structural and functional information on a small chemical compound of the National Cancer Institute chemical database, NSC23766, targeted against the Rho GTPase Rac1 [6] and utilizing a virtual screening strategy using the ZINC database [19], we generated 17 chemically diverse potential Rho GTPase-inhibiting compound formulas, which were then synthesized by SPECS (Delft, Netherlands). Subsequently, all synthesized compounds were tested *in vitro* for solubility characteristics.

### Cytotoxicity assay

Lactate dehydrogenase (LDH) release in cells was assessed with the CytoTox96 Non-Radioactive Cytotoxicity Assay (Promega, Madison, WI) according to the manufacturer’s instructions. Colon cancer cells and S3T3 fibroblasts were seeded in 96-well plates, cultured for 24 h and then incubated with 1–100 μM AZA197 for 24 h. Culture medium was then harvested, centrifuged and supernatants transferred to a 96-well plate. Samples were mixed with freshly prepared substrate mix, incubated protected from light for 30 min at room temperature and after addition of stop solution, absorbance was measured at 490 nm. AZA197 mediated cytotoxicity expressed as LDH release (%) was determined as % Cytotoxicity = [Experimental LDH release (OD490)] ÷ [Maximum LDH release (OD490)].

### Rho GTPase activation assays

Colon cancer cells were seeded in 6-well plates. Cells were incubated with 1, 2, 5 and 10 μM AZA197 for 24 h. Rac1-, Cdc42- and RhoA-activation was then measured using G-LISA (Rac1, Cdc42 and RhoA activation assay, colorimetric format; Cytoskeleton, Denver, CO) according to the manufacturer’s protocol.

### Guanine nucleotide-exchange assay *in vitro*

GEF activity was measured with the RhoGEF Exchange Assay Biochem Kit (Cytoskeleton, Inc., Denver, CO) according to the manufacturer’s instructions. Briefly, fluorescence spectroscopic analysis of N-methylanthraniloyl (mant)-GTP incorporation into purified His-tagged Cdc42 was carried out using a Perkin Elmer EnSpire multimode plate reader at 20°C. Exchange reaction assay mixtures containing 20 mM Tris (pH 7.5), 50 mM NaCl, 10 mM MgCl_2_, 50 μg/ml BSA, 0.8 μM mant-GTP and 1 μM Cdc42 GTPase were prepared in the presence or absence of 10 μM AZA197. After equilibration, assays were placed into sample holders and fluorescence measurements taken every 30 sec at excitation and emission wavelengths of 360 nm and 440 nm, respectively. After five readings (150 sec), Dbs or water (control) was added to 0.8 μM and relative mant fluorescence (λex = 360 nm, λem = 440 nm) readings were taken for a total reaction time of 30 minutes (1800 seconds). Experiments were performed in triplicate.

### Cell proliferation assay

Human SW620 cells were seeded in 96-well plates at a density of 1×10^4^ cells/well in culture medium. Cells were incubated with 1, 2, 5 or 10 μM AZA197. Cell proliferation was determined at 24, 48 or 72 h after treatment using the WST-1 reagent (Roche Diagnostics, Indianapolis, IN) according to the manufacturer’s protocol [20]. Each experiment was repeated three times.

### FACS analysis

Tumor cells were seeded in 6-well plates and allowed to adhere before treatment with 2, 5 or 10 μM AZA197. Cells were then trypsinized, washed in PBS, fixed in 70% ethanol for 1 h at 4°C and subsequently stained in PBS supplemented with 800 μg/ml propidium iodide containing 50 μg/ml RNaseA. 10^4^ events were analyzed on a FACScan flow cytometer (BD Biosciences) with an argon laser tuned to 488 nm.

### Migration assay

Colon cancer cells (1×10^5^ in 1 ml DMEM with 10% FCS) were added to the top of each Boyden migration chamber (8 μm, 12-well plate format; BD Biosciences, Palo Alto, CA). Cells were incubated with 1, 2 and 5 μM of AZA197. After 24 h, medium was removed and membranes were washed twice with phosphate buffered saline (PBS). Cells from the upper side of the membrane were removed with cotton swabs. The membranes were excised using a scalpel, inverted and transferred to a PBS filled tissue culture well. Membranes were then fixed in methanol for 10 min at -20°C. After washing in PBS, membranes were stained with 1 μg/ml 4′-6-Diamidino-2-phenylindole (DAPI) in PBS for 10 min at room temperature and washed again in PBS. Membranes were then embedded in Cityfluor (Cityfluor, Leicester, UK) on glass slides. Representative sectors of migrated colon cancer cells were counted under a fluorescence microscope. Each experiment was performed in triplicate.

### Invasion assay

Colon cancer cells (5x10^4^ in 1 ml culture medium) were added to the top of each BioCoat Matrigel Invasion Chamber (8 μm, 24-well plate format; BD Biosciences) in DMEM supplemented with 0.2% FCS according to the manufacturer’s protocol. As chemoattractant, DMEM containing 10% FCS was added to the lower chamber. AZA197 was then added to 1, 2 and 5 μM. After 24 h, the medium was removed and membranes were washed twice with PBS and stained as described previously for the migration assays. Representative sectors of invaded colon cancer cells were counted under a fluorescence microscope. Each experiment was performed in triplicate.

### Visualization of the actin cytoskeleton and fluorescence microscopy

Human SW620 and HT-29 cells were grown on a chambered coverglass coated with fibronectin/gelatin (0.001% fibronectin/0.2% gelatin in distilled water) in culture medium and were then incubated with 5 or 10 μM AZA197 for 24 h. Cells were then fixed, permeabilized, labelled with Atto-488 phalloidin (Sigma-Aldrich, St. Louis, MO) and counterstained with 4′,6-Diamidino-2-Phenylindole, Dihydrochloride (DAPI, Invitrogen). Fluorescence was observed with a Nikon Eclipse 80i (Tokyo, Japan) microscope equipped with DAPI and fluorescein-isothiocyanate (FITC) filters at 1,000× magnification and images were digitally acquired.

### Western blotting

Colon cancer cells were seeded in 100 mm plates and incubated with 2, 5 and 10 μM AZA197 for 24 h. Cell lysates were prepared [21,22] and 50 μg/lane were separated by 12% SDS-PAGE prior to electrophoretic transfer onto Hybond C super (Amersham Pharmacia Biotech, Buckinghamshire, UK). The blots were probed with antibodies against Cdc42, PAK1, phospho-PAK1 (Ser144)/PAK2(Ser141), ERK1/2, phospho-44/42 ERK1/2 (Thr202/Tyr204), Cyclin D1 (Cell Signaling Technology, Danvers, MA) and α-tubulin (clone B-5-1-2, Sigma-Aldrich) before incubation with horseradish peroxidase–conjugated secondary antibodies (Amersham Pharmacia Biotech). Reversible Ponceau S staining and α-tubulin staining were used as a loading control. Proteins were immunodetected by chemiluminescence (Supersignal-West- Femto, Pierce, Rockford, IL), scanned using FUSION-FX7 (Vilber Lourmat) and quantified by Fusion-CAPT-Software 16.07 (Vilber Lourmat).

### Tumor model

The experiments performed in this study were approved by the Institutional Animal Care and Use Committee at the Vienna Medical University. Pathogen-free, male, 5 week-old athymic *nu/nu* (nude) mice (Charles River, Sulzfeld Germany) were weighed, coded and divided into experimental groups of (*n* = 8) at random. Mice were anesthetized (ketamine hydrochloride/xylazine at 55/7.5 mg/kg i.p.) and 8×10^6^ SW620 cells/100 μl PBS were injected s.c. into the left flank [20,21]. Eight days after cell injection, mice received daily i.p. injections with 100 μg AZA197 in 100 μl 30% DMSO for two weeks, control animals received 100 μl 30% DMSO/day. Tumor volumes were calculated as length × width^2^÷2 using a caliper. All animals were sacrificed on day 22 and tumor weights were assessed.

### Analysis of the effects of AZA197 *in vivo*

On day 22 the animals were sacrificed. Tumors were photographed in situ following removal of the surrounding skin, isolated and weighed. One portion of the tissue was processed for paraffin embedding and serial sections were made. Sections were rehydrated, incubated in 5% H_2_O_2_ to block endogenous peroxidase activity and antigens detected with Ki-67 antibody (tumor proliferation assay; Dako, Glostrup, Denmark) to evaluate the density of proliferating cells [21,22]. Primary antibodies were detected by sequential incubation with biotinylated secondary antibody (Vector Laboratories, Burlingame, CA) and peroxidase conjugated streptavidin (Dako, Glostrup, Denmark), developed with 3, 3′-diaminobenzidine (Vector Laboratories), counterstained with haemalaun, dehydrated and mounted in DPX (Merck, Darmstadt, Germany) and digitalized images were generated.

### Tissue terminal deoxynucleotide transferase–mediated dUTP nick end labeling assay

Histological analysis of nuclei exhibiting DNA fragmentation was used to identify apoptotic cells in paraffin sections of SW620 xenograft tumors by i*n situ* terminal deoxynucleotide transferase–mediated dUTP nick end labeling (TUNEL) with the use of an apoptosis detection kit (In Situ Cell Death Detection Kit, Fluorescein; Roche Diagnostics, Indianapolis, IN) according to the manufacturer’s instructions. The number of TUNEL-positive apoptotic cells was evaluated by fluorescence microscopy. Results are expressed as relative percentage of TUNEL–positive cells per field.

### Analysis of the effects of AZA197 on survival

The survival study was set for 100 days. Mice were treated with AZA197 (*n* = 6) or 30% DMSO in controls (*n* = 6) and were euthanized when moribound.

### Statistical analysis

Data were tested for normality using the Shapiro-Wilk test. Groups were compared by analysis of variance (ANOVA) and by nonparametric analysis (Wilcoxon rank-sum test, Kruskal-Wallis test). All statistical tests were two-sided. The overall survival curves after treatment were analyzed by the Kaplan-Meier survival test. Statistical tests were performed with the use of SPSS software (version 20.0.2; SPSS, Chicago, IL). Data are expressed as means ± SD. *P* values of < 0.05 were considered to indicate statistical significance.

## Results

### Identification of AZA197

**Figure 1:**
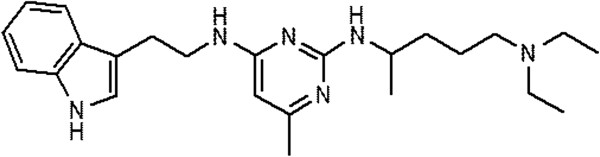
Structural formula of AZA197 (N2-(4-Diethylamino-1-methyl-butyl)-N4-[2-(1H-indol-3-yl)-ethyl]-6-methyl-pyrimidine-2,4-diamine).

An *in vitro* screen of small molecule inhibitors based on modifications of NSC23766 to identify inhibitory compound activity identified the structure (N2-(4-Diethylamino-1-methyl-butyl)-N4-[2-(1H-indol-3-yl)-ethyl]-6-methyl-pyrimidine-2,4-diamine), named AZA197 (Figure 1) to have strong inhibitory activity in SW620 colon cancer cells.

### Cytoxicity evaluation of AZA197

**Figure 2:**
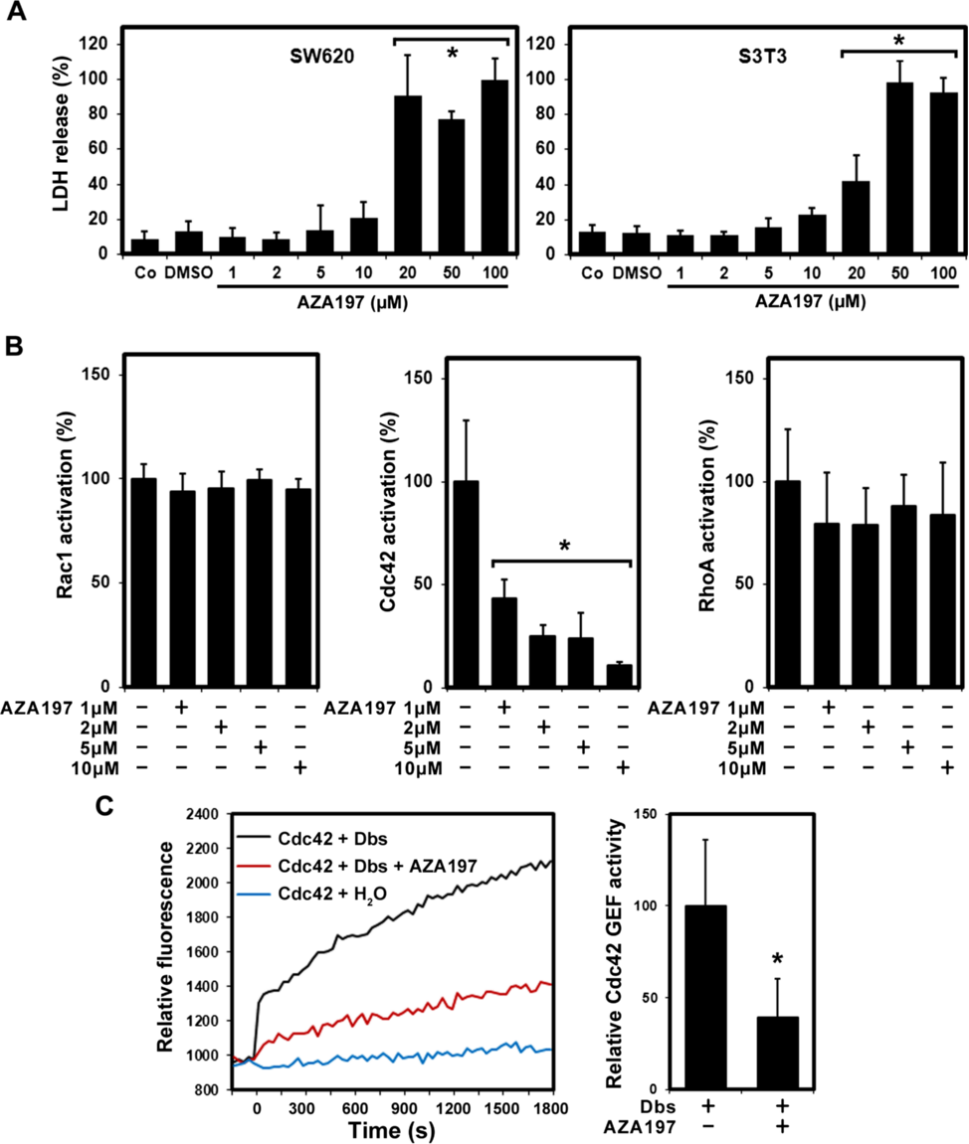
**Compound AZA197 promotes LDH release and inhibits Cdc42 activation. A** Cytotoxicity was assessed by LDH release in SW620 colon cancer cells and S3T3 fibroblasts after 24 h exposure to AZA197 (1–100 μM). Co, untreated control; DMSO, solvent control; *, significantly different from untreated control. **B** Rac1, Cdc42 and RhoA activation in SW620 colon cancer cells after incubation (24 h) with different concentrations of compound AZA197. AZA197 suppresses Cdc42 activity in colon cancer cells in a dose-dependent manner. Means of three independent experiments are shown. *, significantly different from untreated control. **C** AZA197 inhibits Cdc42 GDP/GTP exchange stimulated by GEF. AZA197 significantly inhibits the GEF activity of Dbs on Cdc42 assessed by mant fluoresence. Experiments were performed in triplicate and data from one representative experiment are presented. The bar chart shows the statistical analysis of relative Cdc42 GEF activity from these measurements. *, significantly different from untreated controls.

The cytotoxic effect of different concentrations of AZA197 was examined by LDH release in SW620 colon cancer cells, HT-29 colon cancer cells and S3T3 fibroblasts. DMSO control samples were included to assess potential cytotoxic effects of the compound solvent. In both cancer cells and fibroblasts, a similar AZA197 toxicity profile from 1–100 μM was observed (Figure 2A for SW620 and S3T3; Additional file 1: Figure S1A for HT-29). LDH release in cells exposed to DMSO ranged from 12.5% in S3T3 fibroblasts, 12.7% in HT-29 cells to 13.2% in SW620 cells. The LDH release profiles in all investigated cells exposed to AZA197 up to 10 μM was comparable to solvent control cultures. At higher AZA197 concentrations of 20, 50 and 100 μM, significantly increased levels of LDH release were observed in all cell lines investigated with a 9-fold increase in SW620 cells (*P* < 0.001) and 3-fold increases in HT-29 cells (*P* < 0.04) and S3T3 fibroblasts (*P* < 0.001) at 20 μM (Figure 2A; Additional file 1: Figure S1A). In addition, bright-field microscopy did not reveal any morphological features suggestive of cytotoxicity, such as membrane blebbing, at concentrations up to 10 μM (data not shown). However, there was a drastic change in cell morphology at concentrations above 10 μM which included blebbing and evidence of nuclear fragmentation.

These data suggest that low plasma membrane damage occurs independently of the cell type after 24 h of exposure to AZA197 at concentrations up to 10 μM as evidenced by low intracellular LDH release. The cytotoxic responses in both fibroblasts and cancer cells above 20 μM prompted us to use concentrations up to 10 μM for further *in vitro* experiments analyzing the anti-tumor effects of AZA197.

### AZA197 treatment inhibits Cdc42 activity in colon cancer cells

The effect of AZA197 on the activity of Rac1, Cdc42 and RhoA GTPases was comparatively assessed in G-LISA assays. We first examined Rac1 activation in SW620 colon cancer cell lysates. Treatment with 1, 2, 5 or 10 μM AZA197 did not affect Rac1 activity (Figure 2B left panel). AZA197 inhibited Cdc42 in a dose-dependent manner in SW620 cells. AZA197 reduced Cdc42 activity significantly by 56.7% (*P* = 0.024), 75.2% (*P* = 0.014), 76.0% (*P* = 0.035) and 89.3% (*P* = 0.011) at 1, 2, 5 and 10 μM, respectively, compared to untreated controls (Figure 2B central panel). In contrast, RhoA activity was not significantly affected by AZA197 treatment in SW620 cells (Figure 2 right panel). AZA197 also dose-dependently and significantly down-regulated Cdc42 activity in HT-29 colon cells by 18% (1 μM, *P* = 0.048), 48.5% (2 μM, *P* = 0.011), 52.9% (5 μM, *P* = 0.014) and 61.0% (10 μM, *P* < 0.001) as shown in Additional file 1: Figure S1B. Similar to SW620 cells, AZA197 treatment caused no suppression of Rac1 or RhoA activity in HT-29 cells (Additional file 1: Figure S1B). These results indicate that AZA197 specifically and significantly down-regulates Cdc42 activity in the human SW620 and HT-29 colon cancer cell lines with no effects on Rac1 or RhoA GTPase family members.

### Compound AZA197 inhibits Cdc42–GEF interaction *in vitro*

Since AZA197 specifically inhibits Cdc42 activity, we hypothesized that AZA197 can act as a Cdc42–GEF interaction-specific small-molecule inhibitor. To determine whether AZA197 is active in inhibiting the GEF-stimulated guanine nucleotide-exchange reaction of Cdc42, an *in vitro* nucleotide-exchange assay was performed. The GEF activity of Dbs on Cdc42 was used as a positive control and water as a negative control. As shown in Figure 2C, mant fluorescence intensity increased dramatically when purified Dbs domains were added to Cdc42. Incubation with AZA197 reduced the exchange activity of Dbs domains on Cdc42 by approximately 61% compared to the GEF activity of Dbs on Cdc42 (*P* = 0.034). These data indicate that AZA197 is able to block the nucleotide exchange of Cdc42 thereby preventing Cdc42 activation by disrupting the interaction of Cdc42 with GEFs *in vitro*.

### AZA197 suppresses cell proliferation in SW620 cells

**Figure 3:**
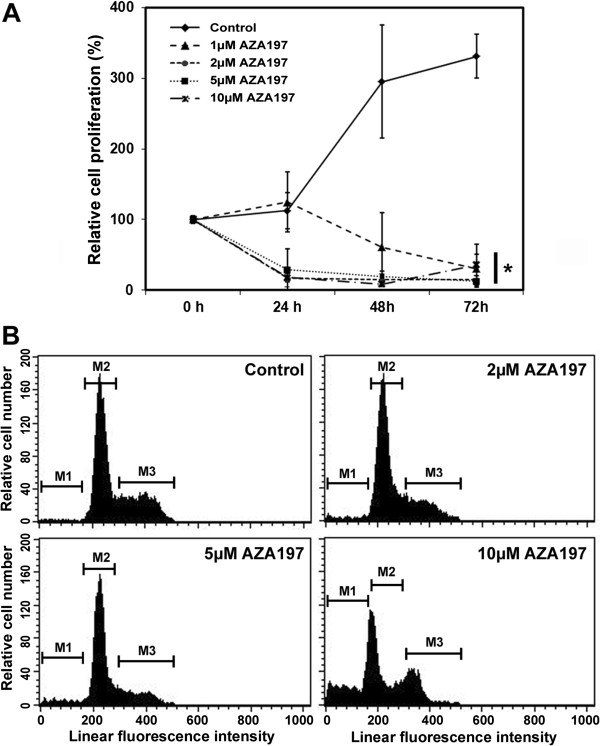
**Effects of Cdc42 inhibition by AZA197 on cell proliferation in SW620 colon cancer cells. A** Relative density of cancer cells up to 72 h following treatment with 1, 2, 5, and 10 μM compound AZA197 was measured using the WST-1 cell proliferation assay. AZA197 suppresses SW620 colon cancer cell proliferation in a dose-dependent manner. Means of three independent experiments are shown. *, significantly different from control. **B** Cellular DNA content was analyzed by flow cytometry after staining with propidium iodide. Representative flow cytometry histograms showing SW620 cells treated with 2, 5 or 10 μM AZA197 for 24 h. Control cells received no treatment. AZA197 dose-dependently reduces the number of cells in the S and G_2/M_ phases (M3) and increases the number of cells with sub G_0_/G_1_ DNA content (M1) characteristic of apoptosis.

Activation of Cdc42 stimulates many signaling cascades that alter cellular processes such as proliferation and migration [15]. To test whether AZA197 affects colon cancer cell proliferation, we treated human SW620 and HT-29 cells with different concentrations of compound and determined the increase in mass of cellular protein for up to 72 h. Both SW620 and HT-29 cell proliferation were significantly reduced after 72 h incubation with 1, 2, 5 and 10 μM of compound compared to untreated control cells (*P* < 0.001) (Figure 3A for SW620, and Additional file 2: Figure S2 for HT-29). Treatment with AZA197 suppressed SW620 and HT-29 cell proliferation in a dose-dependent manner.

To test whether AZA197 has an influence on the cell cycle, we treated SW620 colon cancer cells with different compound concentrations. Treatment with AZA197 reduced cell proliferation (reduction in S and G_2/M_ phases, M3) and increased the number of apoptotic cells (sub G_0_/G_1_ peak, M1) in a dose-dependent manner (Figure 3B). These data indicate that AZA197 reduces colon cancer cell proliferation associated with increased apoptosis.

### AZA197 reduces the migration and invasion of colon cancer cells

**Figure 4:**
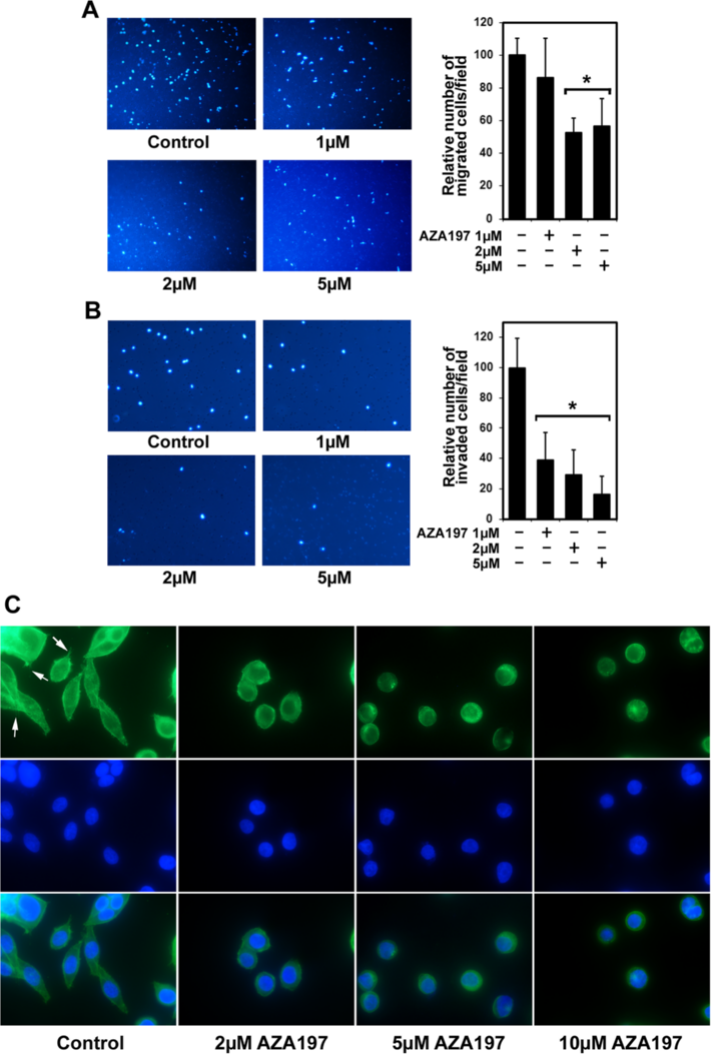
**Cdc42 blockade reduces colon cancer cell migration, invasion and affects actin cytoskeleton reorganisation. A** Representative images of migrated SW620 colon cancer cells from an *in vitro* migration assay are shown. Colon cancer cells were treated with 1, 2 or 5 μM AZA197 for 24 h and migrated cancer cells quantified subsequently by *in vitro* migration assays. Data were collected from five individual consecutive fields of view (40x) from three replicate Boyden chambers. *, significantly different from controls. **B** The invasive capacity of SW620 cells was determined in matrigel invasion assays. Invaded SW620 cells were quantified from five individual consecutive fields of view (100x) from three replicate chambers. *, significantly different from control. **C** Effect of AZA197 treatment on cell morphology, filopodia formation and actin reorganization. SW620 colon cancer cells were plated on fibronectin/gelatin coated cell culture chambers and incubated with 2, 5 and 10 μM AZA197 for 24 h. Paraformaldehyde fixed cells were stained with Atto-488 phalloidin (F-actin, green) to visualize the polymerized actin cytoskeleton and filopodia and subsequently counterstained with DAPI (blue) and photographed (magnification, x1000). Arrows indicate filopodia. AZA197 leads to changes in cellular morphology and suppresses filopodia formation.

Rho GTPases such as Cdc42 can also play an essential role in tumor cell migration [23]. We therefore examined the effect of AZA197 on migration of SW620 cells in a transwell assay. Treatment of cells with 1 μM compound for 24 h only moderately reduced cancer cell migration compared to untreated controls (Figure 4A). Treatment of cells with 2 or 5 μM AZA197 significantly reduced cancer cell migration by 47.4 ± 8.8% (*P* < 0.05) and 43.5 ± 17% (*P* < 0.05), respectively, compared to untreated controls (Figure 4A). Similarly, AZA197 significantly reduced cancer cell migration in a dose-dependent manner up to 77.1% (*P* < 0.024) in HT-29 colon cancer cells (Additional file 3: Figure S3A). These results indicate a role for AZA197 in blocking Cdc42-dependent migration of SW620 colon cancer cells.

Since migration and invasion of cancer cells are key steps in tumor metastasis, we assessed the effects of AZA197 on SW620 and HT-29 cancer cell invasion in a matrigel cell invasion assay. As shown in Figure 4B, treatment of SW620 cells with 1, 2 and 5 μM compound AZA197 for 24 h significantly decreased SW620 invasion by 61.3 ± 18%, 71.0 ± 16.6% and 83.9 ± 12.4% (*P* < 0.003), respectively, compared to untreated controls. Similarily, AZA197 also significantly decreased invasion of HT-29 cells at corresponding concentrations up to 84.6% (*P* < 0.005) compared to controls (Additional file 3: Figure S3B).

Together, these results suggest that AZA197 is highly effective in preventing SW620 and HT-29 colon cancer cell migration and invasion in a dose-dependent manner.

### Morphological and actin cytoskeleton changes in AZA197 treated colon cancer cells

Cdc42 is also crucial in the formation of filopodia, which are important in the motility of cancer cells [24]. We therefore investigated the effect of AZA197 on colon cancer cell morphology with phalloidin that specifically stains the polymerized actin cytoskeleton. In subconfluent SW620 controls, elongated cell morphology was observed and a high number of filopodia identified (Figure 4C). Treatment with AZA197 at 2, 5 and 10 μM caused cells to become rounded and filopodia formation was dramatically diminished after 24 h (Figure 4C). HT-29 cells displayed spreading morphology and a normal filamentous actin distribution in the surface protrusions but cells treated with 2, 5 and 10 μM AZA197 exhibited diminished cell spreading, a rounded cell morphology with no surface protrusions and formation of submembranous cortical actin (Additional file 3: Figure S3C).

These results suggest that treatment of colon cancer cells with AZA197 results in an alteration of the actin cytoskeleton and cell morphology in colon cancer cells and reduces filopodia formation in SW620 cells.

### The PAK1 signaling pathway is down-regulated by AZA197 treatment in colon cancer cells

**Figure 5:**
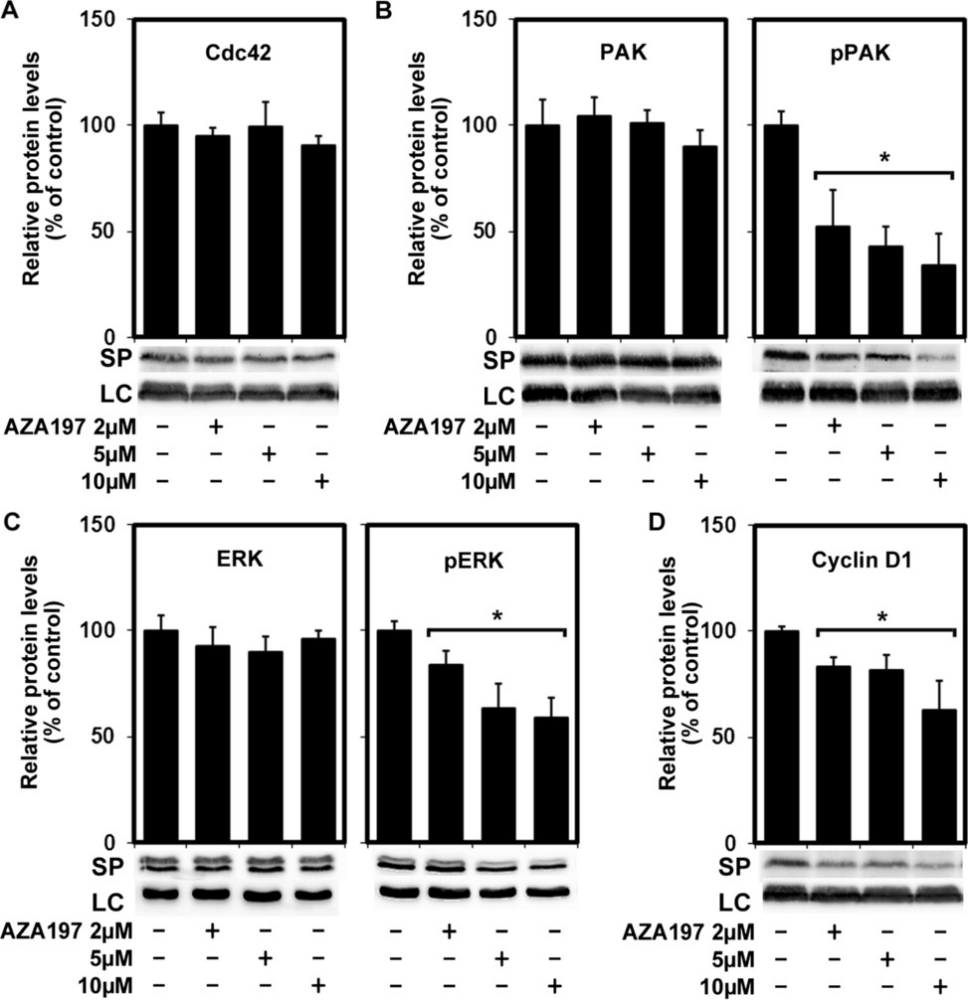
**Analysis of AZA197-signal transduction effectors in SW620 colon cancer cells. A** Cdc42 levels were not changed in SW620 cells treated with AZA197 compared to untreated cells. Means of 3 independent experiments are shown. **B,C** Analysis of PAK1 **(B)** and ERK **(C)** phosphorylation in SW620 colon cancer cells after AZA197 treatment. Representative Western blot images and quantification of immunoblots stained with PAK1, phospho-PAK1/2 (pPAK) and ERK, phospho-ERK (pERK) antibodies before and after treatment with 2, 5, and 10 μM AZA197 for 24 h. Cdc42 blockade reduces PAK1 and ERK phosphorylation in SW620 cells (mean of 3 independent experiments) without affecting total protein levels. *, significantly different from control. **D** Analysis of CyclinD1 expression in SW620 colon cancer cells following AZA197 treatment. Representative Western blot images and quantification of immunoblots stained with CyclinD1 antibody before and after treatment with 2, 5, and 10 μM AZA197 for 24 h. CyclinD1 levels were reduced following AZA197 treatment of SW620 cells (mean of three independent experiments). LC, loading control; SP, specific protein; *, significantly different from control.

To analyze whether AZA197 affects Cdc42 protein expression, we measured Cdc42 protein levels by Western blot analysis. In both SW620 (Figure 5A) and HT-29 (Additional file 4: Figure S4A), Cdc42 protein levels were not affected by treatment with different concentrations of AZA197 suggesting that AZA197 does not affect levels of Cdc42 protein expression.

Group I p21-activated kinases (PAKs) have been implicated in colon cancer cell transformation in expression and functional studies and are important effectors of the small GTPase Cdc42 [25]. To analyze signaling pathways that could mediate the effects of AZA197 on Cdc42 inhibition, we examined the activity of the downstream effector PAK by evaluating PAK phosphorylation in SW620 and HT-29 colon cancer cells following AZA197 treatment. Although no reduction in PAK expression was seen (Figure 5B, left panel), PAK1/2 phosphorylation at serine 144/141, which maintains the kinase activity of PAKs [26], was dose-dependently significantly reduced by 47.7 ± 6.5% (2 μM), 57.2 ± 17.3% (5 μM) and 66.2 ± 15.3% (10 μM) (*P* < 0.01) after treatment with 2, 5 and 10 μM AZA197 for 24 h in SW620 cells compared to untreated cells (Figure 5B), respectively. Similarly, PAK1/2 phosphorylation was also dose-dependently and significantly reduced up to 72.8 ± 15.8% (*P* < 0.014) on AZA197 treatment of HT-29 cells without influencing total PAK protein expression (Additional file 4: Figure S4B), indicating that Cdc42 inhibition blocks the PAK1 signaling pathway in these colon cancer cells. These findings suggest that AZA197 mediated Cdc42 inhibition is associated with reduced PAK1/2 phosphorylation.

To identify further downstream Cdc42 effectors affected by AZA197 treatment, we analyzed MAPK activity using phospho-specific antibodies. ERK activity is decreased by PAK1 deactivation leading to decreased cell proliferation, migration/invasion and survival in colon cancer [27]. Our data show that Cdc42 inhibition by AZA197 for 24 h led to a significant dose-dependent inhibition of phospho-ERK levels by 16.1 ± 4.6% (2 μM), 36.7 ± 12% (5 μM) and 40.2 ± 9.1% (10 μM) (*P* < 0.05) in SW620 cells, whereas total ERK protein levels were not affected by AZA197 treatment (Figure 5C). In HT-29 cells, phospho-ERK levels were also significantly reduced by 29.1 ± 15.7% (2 μM), 38.2 ± 3.5% (5 μM) and 44.4 ± 6.3% (10 μM) by AZA197 treatment (*P* < 0.04) compared to untreated cells (Additional file 4: Figure S4C). In contrast, levels of activated p38 and JNK did not show any significant changes in response to AZA197 treatment in colon cancer cells (data not shown).

We then tested the effect of AZA197 on the cell cycle regulatory protein Cyclin D1, since Rho family GTPases have been shown to be essential for the Cyclin D1 up-regulation associated with G1 to S phase transition. In addition, PAK has been shown to play a role in upregulation of Cyclin D1 involving activation of ERK kinase [28]. In SW620 colon cancer cells treated with 2, 5, and 10 μM AZA197 for 24 h, Cyclin D1 protein expression decreased significantly by 16.8 ± 2.2% (2 μM), 18.6 ± 4.5% (5 μM) and 37.1 ± 14.1% (10 μM) (*P* < 0.05) compared to untreated controls as shown in Figure 5D. In HT-29 cells, Cyclin D1 protein expression was significantly reduced when treated with 5 μM (22 ± 5.5% reduction) and 10 μM (20.8 ± 9.9% reduction) (*P* < 0.049) but not with 2 μM AZA197 (Additional file 4: Figure S4D).

These results suggest targeting Cdc42 with the small molecule inhibitor AZA197 in colon cancer cells can effectively modulate PAK/ERK signaling interfering with Cyclin D1 expression to affect colon cancer cell proliferation.

### AZA197 suppresses primary colon cancer growth and prolongs animal survival

**Figure 6:**
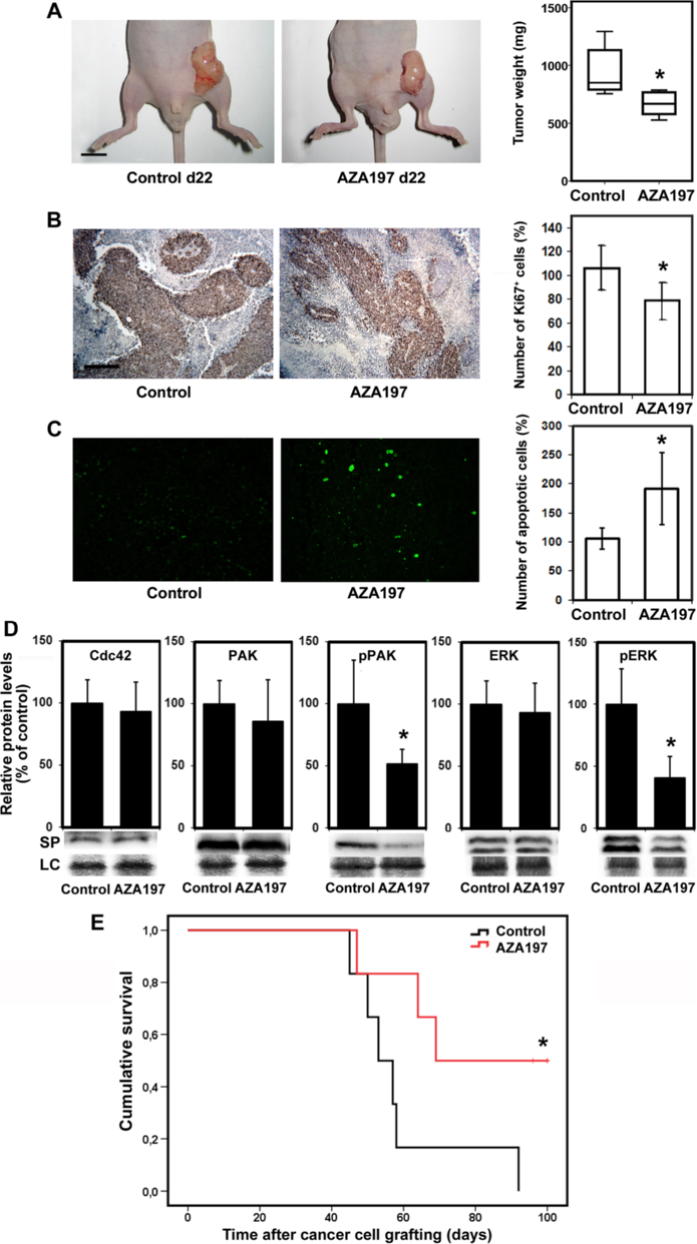
**Cdc42 blockade by AZA197 suppresses tumor growth and prolongs animal survival. A** Left panel: representative images of human SW620 tumor xenografts on day 22 from mice treated with solvent (Control) or 100 μg/day AZA197 (AZA197 d22) (bar = 10 mm). Right panel: AZA197 significantly suppresses tumor weight of human colon cancer xenografts in mice. Data are shown as mean tumor weights on day 22. *, significantly different from control on day 22 (*P* = 0.006). **B** Effect of AZA197 on proliferation in SW620 xenografts. Left panel: representative immunohistochemistry images of tumor tissue sections from mice treated with solvent (Control) or 100 μg/day AZA197 on day 22 stained with Ki-67 antibody (bar = 200 μm). Right panel: Quantitative histomorphometric analysis of Ki-67-positive, proliferating tumor cells. *, significantly different from control (*P* = 0.046). **C** Effect of AZA197 on apoptosis in SW620 xenografts. TUNEL labeling of cells undergoing DNA fragmentation: Left panel: representative images of tumor tissue sections from mice treated with solvent (Control) or 100 μg/day AZA197 on day 22 (bar = 50 μm). Right panel: Quantitative histomorphometric analysis of TUNEL-positive, apoptotic tumor cells. *, significantly different from control (*P* = 0.035). **D** Analysis of total Cdc42 protein expression and levels of PAK1 and ERK phosphorylation in SW620 colon cancer tissue on day 22 from mice treated with solvent (Control) or 100 μg/day AZA197 (AZA197 d22). Representative Western blot images and quantification of immunoblots stained with Cdc42, PAK1 and phospho-PAK1/2 (pPAK), ERK and phospho-ERK antibodies. Cdc42 blockade by AZA197 reduces PAK1/2 and ERK phosphorylation in SW620 tumor tissue. *, significantly different from control. **E** Effect of AZA197 treatment on animal survival in SW620 colon cancer bearing mice. Kaplan-Meier survival curves for controls and AZA197 treated mice. AZA197 significantly prolongs animal survival.*, significantly different to control (*P* = 0.042).

To analyze whether treatment with AZA197 can modulate tumor growth *in vivo*, we treated mice bearing human SW620 colon cancer xenografts with AZA197 or vehicle as controls. To assess treatment modalities *in vivo*, we initially assessed AZA197 stability *in vitro* (data not shown) and cycled treatment daily for two weeks to guarantee continuous delivery of the compound. At the beginning of treatment on day 8, mice developed tumor xenografts of comparable size. On day 22, the mean tumor weight was significantly (*P* = 0.006) reduced in mice treated with AZA197 (676.7 ± 106 mg) compared to control mice (968 ± 208 mg) and treatment was well tolerated (Figure 6A). To compare the proliferation and apoptotic rate of untreated tumors and tumors treated with AZA197, tumor sections were stained for expression of Ki-67 and DNA fragmentation by TUNEL assays, respectively. In accordance with the tumor weight reduction findings, treatment with AZA197 decreased the number of Ki-67-positive cells in tumors based on counting 20 randomly selected microscopic fields by 27.4 ± 14.2% (*P* = 0.046) in AZA197-treated tumors, suggesting an anti-proliferative effect for AZA197 (Figure 6B). Moreover, AZA197-treated tumors showed increased numbers of apoptotic cells as assessed by positive staining for TUNEL compared with untreated controls. Based on the counting of randomly selected microscopic fields, the number of apoptotic cells was increased by 80.6 ± 58.3% (*P* = 0.035) from controls to AZA197-treated tumors (Figure 6C). Western blotting of isolated tumor tissue indicated that AZA197 treatment does not change Cdc42 and total PAK and ERK expression. Phospho-PAK1 expression in tumors treated with AZA197 was significantly reduced by 48.5 ± 11.4% (*P* = 0.027) compared to untreated controls (Figure 6D). Similarly, in tumors treated with AZA197, phospho-ERK levels decreased significantly by 59.2 ± 17.1% (*P* = 0.003) compared to untreated controls (Figure 6D). These data show that the PAK-ERK signaling pathway is a downstream target of the small molecule inhibitor AZA197 in SW620 colon cancer tissue confirming our findings *in vitro*.

In mice bearing colon cancer xenografts, the median time to death in the control group was 53 days and all mice died between 45 and 92 days after tumor cell grafting. However, survival was significantly increased in mice following AZA197 treatment compared to control mice (*P* = 0.042) and the median time to death was 69 days. On day 100 (at the end of the experiment), all animals in the control group were deceased whereas 50% of AZA197-treated mice were still alive (Figure 6E). Control mice that died on days 45, 57 and 58 had tumor weights of 3455, 4582 and 4810 mg, respectively, whereas mice in the AZA197 treatment group at comparable time points at days 47 and 64 had tumors of 2897 and 3768 mg, respectively, showing that AZA197 treatment results in decreased tumor weight even after the end of treatment on day 22. Together, these data indicate that AZA197 slows primary tumor growth of human SW620 colon cancer xenografts in mice and improves animal survival.

## Discussion

Significant progress has been achieved in deciphering the molecular events associated with the onset of colorectal cancer and molecular analyses are becoming mainstream in planning the management of advanced colorectal cancer with tailored therapies. Although new, targeted therapies have become available in recent years, some patients are resistant to the clinical benefits of these agents which have only a modest impact on disease. In advanced colorectal cancer patients with mutated KRAS, for example, targeted therapies have provided no benefit showing a clear need to establish new therapeutic strategies [29-31].

Although a recent study has shown that a strong decrease in Cdc42 and Rac1 activity in combination with ROCK inhibition is clearly associated with increased colon cancer invasiveness [32], data from previous studies addressing the molecular mechanisms underlying colon cancer progression suggested that Rho family members including Cdc42 may play a critical role in promoting colon cancer progression [15]. Cdc42 is overexpressed in a number of human cancers and may be involved in the promotion of tumorigenesis and Cdc42 activity has been implicated in the invasive phenotype which characterizes tumor metastasis [15]. Analyses of human colorectal cancer specimens identified a high incidence of Cdc42 overexpression [18] and showed that presence of Cdc42 target proteins could be readily detected in tumors from human colorectal cancer patients, providing a screening tool for both enrolling patients in future clinical trials and evaluating the outcome of such trials. In the same study, Cdc42 overexpression in SW620 cancer cells down-regulated the potential tumor suppressor gene ID4 [18], further indicating that Cdc42 may play a role in the development of colon cancer and is a suitable target for intervention in patients with this disease.

Based on these findings, we hypothesized that inhibition of Cdc42 might be effective for the treatment of colorectal cancer. We therefore designed the small molecule Cdc42 inhibitor AZA197 and show that inhibition of Cdc42 activity with AZA197 acts to reduce tumor growth and significantly improve animal survival in SW620 cells which are a model of KRAS mutant colon cancer xenografts [33]. Assays *in vivo* and *in vitro* suggest that inhibition of cell proliferation and induction of apoptosis were the main mechanisms by which AZA197 exerts antitumor effects.

Other Cdc42 modulators such as CID-2950007 [34], secramine [35] and ZCL278 [36] inhibit Cdc42 by different mechanisms. ZCL278 targets the interaction of Cdc42 with a specific Cdc42-GEF intersectin [36], while secramine inhibits Cdc42 activation in a Rho GDI-dependent manner [35]. The Rac-inhibitor NSC23766 inhibits Rac activation by blocking only some of the GEFs that activate Rac1 [6], highlighting the complexity of cellular pathways regulating the activation status of RhoGTPases. In general, many of the described RhoGTPase inhibitors lack specificity. Thus it is possible, that compound screening of modifications of a known inhibitor, such as with the Rac1-GEF inhibitor NSC23766 presented here, can result in the identification of an inhibitor that could affect the activation status of another RhoGTPase which was not predicted by mechanistic *in silico* analysis of binding to the RhoGTPase structure. Since the function of RhoGTPases, including Cdc42, is controlled by multiple upstream regulators and downstream effectors which could be affected by compounds, the efficacy of an inhibitor may depend on the cellular context and effectors expressed. In this context, it is important to mention that, although our data indicate that AZA197 inhibits Cdc42–GEF interaction *in vitro*, analysis of the crystal structure of Cdc42 bound to AZA197 would be necessary to confirm interaction with the region where GEFs associate with Cdc42. Such data would also allow prediction of compound efficacy based on cell type specific expression of GEFs. To analyze AZA197 specificity for Cdc42 inhibition, we tested the effects of AZA197 on inhibition of the Rho GTPase family members Rac and Rho, which also play a role in colon cancer. Studies of the Rho GTPase family member Rac in SW620 cells, genetically modified to either overexpress or lack Rac1 expression, suggested that Rac1 also plays a major role in colorectal adenocarcinoma progression [9]. Rac proteins are overexpressed in various tumors and Rac-dependent cell signaling has been shown to be important for malignant transformation [12]. Our data show that AZA197 does not inhibit Rac activity in SW620 colon cancers. Thus, inhibition of Cdc42-activity alone without affecting Rac-activity could lead to a potent suppression of colon cancer growth and increased survival rates. However, since the Rho GTPases, including Cdc42, are involved in the regulation of many normal cellular functions in a variety of cell types, it is possible that toxicity will limit inhibition of RhoGTPase activity in patients, although inhibition of Cdc42 by AZA197 was well tolerated in the tested context.

Like Cdc42 and Rac, high protein expression levels of the Rho GTPase RhoA appears to be a frequent event in different types of human tumors, including colon cancer [10] and increased RhoA activity correlates with poor prognosis and recurrence in hepatocellular carcinoma [37]. Even though RhoA may be involved in colon cancer progression, our data reveal that RhoA is not suppressed by AZA197 treatment and thus is not a target for AZA197. Unlike RhoA, RhoB is often down-regulated in human tumors and expression inversely correlates with tumor aggressiveness. This can be explained by its potential role as a tumor suppressor and RhoB levels are attenuated commonly during malignant progression [38]. In line with this, we did not detect active RhoB in control or AZA197 treated colon cancer cells, consistent with the general aggressive behavior of these cells (data not shown).

Cdc42 plays an important role in cytoskeleton organization and reducing Cdc42 activity with AZA197 resulted in a loss of filopodia formation and significantly reduced colon cancer cell cell migration and invasion capacity. Data from patients showing that Cdc42 is overexpressed with high incidence in colorectal adenocarcinoma biopsies [18] and the findings in this study, support the notion that Cdc42 inhibition could be used as a therapeutic strategy to fight colorectal cancer. This is supported by a report suggesting that active Cdc42 can enhance colorectal cancer cell migration and invasion [39]. Furthermore, expression of constitutively active Cdc42 significantly increased filopodia formation and cell spread in colorectal cancer cells, which is in line with our findings [39]. Moreover, the finding that inhibition of Cdc42 results in loss of elongated, mesenchymal morphology [40], which we also observed following AZA197 treatment, further strengthens the function of AZA197 as a Cdc42 inhibitor and the tumor promoting role of Cdc42 in colon cancer. However, activation of Cdc42 can induce cell adhesion [41] and it has been recently shown that activated Cdc42 increases SW480 colorectal cancer cell adhesion, migration and invasion [39]. It is therefore possible that AZA197 inhibition of Cdc42 also affects cell adhesion in addition to impairment of colon cancer cell proliferation, migration and invasion.

PAK1 is a main downstream effector of the Rho GTPases Rac1 and Cdc42 [42]. Overexpression of PAK1 has been detected in colorectal cancer and PAK1 expression closely correlated with the aggressive progression of colorectal cancer [17,43]. A recent study showed that PAK1-dependent MAPK pathway activation is required for colorectal cancer cell proliferation. PAK1 knockdown decreased proliferation and delayed the G_1_/S cell-cycle transition and increased apoptosis *in vivo* and *in vitro*[44]. In line with these findings, we observed significant down-regulation of the activation of PAK1 and ERK associated with decreased proliferation following AZA197 treatment in SW620 cancer cells *in vitro* and in SW620 cancer tissue. Additionally, Cdc42 inhibition by AZA197 resulted in increased apoptosis *in vivo* and *in vitro*. Moreover, colon cancer cells overexpressing PAK1 have higher migration rates, whereas down-regulation of PAK1 significantly reduces cell migration [17]. This is in line with our findings of reduced SW620 cancer cell migration following AZA197 treatment. Furthermore, the ERK-dependent pathway is required in PAK1-mediated colon cancer cell migration and invasion [17]. Consequently, the observed down-regulation of the Cdc42-PAK1 signaling pathway could therefore constitute the major effector pathway of AZA197 in colon cancer.

However, there are some limitations to the interpretation of the potential effects of AZA197 on cell proliferation and cancer cell migration and invasion in this study. Our data in SW620 cells suggest that AZA197 may impact cancer cell viability at concentrations that inhibit Cdc42, cell proliferation and actin cytoskeletal changes in SW620 cells. Impaired cell viability may be expected because in addition to regulation of cell migration and invasion, Cdc42 and the downstream signaling mediator PAK1 have also been implicated in regulation of the cell cycle, thereby affecting cell survival and apoptosis [1,12,28], which is in line with our findings in SW620 cells. In contrast, in HT-29 cancer cells, viability and proliferation were not affected by AZA197 at concentrations that significantly inhibit Cdc42 activity as well as cancer cell migration and invasion. Moreover, at concentrations that inhibit Cdc42-mediated morphological changes, we do not see significant effects of AZA197 on cell viability in HT-29 cells. These findings rather suggest cell line-dependent variations in AZA197 effects than a general unspecific effect of AZA197 on cell viability. Importantly, our data also demonstrate that AZA197 does not affect the viability of fibroblasts at effective concentrations indicating AZA197 to be a viable, anti-cancer therapeutic agent with only minor toxicity to normal cells. Our studies in athymic nude mice revealed no changes in body weight or gross indications of toxicity (data not shown). It may therefore be expected that use of AZA197 as an anti-cancer therapeutic in colon cancer would result in a varying response to the compound depending on the specific genetics of the cancer cells.

## Conclusions

In summary, the present study describes a novel small molecule inhibitor which can be used to effectively inhibit the Rho GTPase Cdc42 in the treatment of KRAS mutant colorectal cancers. We provide evidence that Cdc42 inhibition by AZA197 treatment suppresses proliferative and pro-survival signaling pathways via PAK1-ERK signaling and reduces colon cancer cell migration and invasion. Furthermore, we show that systemic AZA197 treatment *in vivo* reduces primary tumor growth and prolongs survival in KRAS mutant colon cancer xenograft-bearing mice. We propose that therapy targeting Rho GTPase Cdc42 signaling pathways may be effective for treatment of patients with advanced colon cancer overexpressing Cdc42 and particularly those with KRAS-mutant disease.

## Abbreviations

GEFs: Guanine nucleotide exchange factors; Cdc42: Cell division control protein 42; PAK: p21-activated protein kinase; ERK: Extracellular-signal regulated kinase; JNK: c-Jun N-terminal kinase; MAPK: Mitogen-activated protein kinase; DMEM: Dulbecco’s modified Eagle’s medium; DAPI: 4′-6-Diamidino-2-phenylindole; PBS: Phosphate buffered saline; FCS: Fetal calf serum; DMSO: Dimethyl sulfoxide; TUNEL: Terminal deoxynucleotide transferase–mediated dUTP nick end labeling.

## Competing interests

The authors declare that they have no competing interests.

## Authors’ contributions

KZ, DA, SA are responsible for the study design. KZ, SG, TL and DA performed the experiments and drafted the manuscript. KZ, SG and TL collected the data. KZ, SG, TL and DA participated in the data analysis and interpretation. All authors read and approved the final manuscript.

## Authors’ information

This study was supported by a PRIZE grant (No. FA 10240013) and by the Herzfelder’sche Familienstiftung.

## Supplementary Material

Additional file 1: Figure S1 Compound AZA197 promotes LDH release and inhibits Cdc42 activation. **A** Cytotoxicity was assessed by LDH release in HT-29 colon cancer cells after 24 h exposure to AZA197 (1–100 μM). Co, untreated control; DMSO, solvent control; *, significantly different from untreated control. **B** Rac1, Cdc42 and RhoA activation in HT-29 colon cancer cells after incubation (24 h) with different concentrations of compound AZA197. AZA197 suppresses Cdc42 activity in colon cancer cells in a dose-dependent manner. Means of three independent experiments are shown. *, significantly different from untreated control. Click here for file

Additional file 2: Figure S2 Effects of Cdc42 inhibition by AZA197 on cell proliferation in HT-29 colon cancer cells. A Relative density of cancer cells up to 72 h following treatment with 1, 2, 5 and 10 μM AZA197 was measured using the WST-1 cell proliferation assay. AZA197 suppresses HT-29 colon cancer cell proliferation in a dose-dependent manner. Means of three independent experiments are shown. *, significantly different from control. Click here for file

Additional file 3: Figure S3 Cdc42 blockade reduces colon cancer cell migration, invasion and affects actin cytoskeleton reorganization. **A** Representative images of migrated HT-29 colon cancer cells from an *in vitro* migration assay are shown. Colon cancer cells were treated with 1, 2 or 5 μM AZA197 for 24 h and migrated cancer cells quantified subsequently by *in vitro* migration assays. Data were collected from five individual consecutive fields of view (40x) from three replicate Boyden chambers. *, significantly different from control. **B** The invasive capacity of HT-29 cells was determined in matrigel invasion assays. Invaded HT-29 cells were quantified from five individual consecutive fields of view (100x) from three replicate chambers. *, significantly different from control. **C** Effect of AZA197 treatment on cell morphology, filopodia formation and actin reorganization. HT-29 colon cancer cells were plated on fibronectin/gelatin coated cell culture chambers and incubated with 2, 5 and 10 μM AZA197 for 24 h. Paraformaldehyde fixed cells were stained with Atto-488 phalloidin (F-actin, green) to visualize the polymerized actin cytoskeleton and filopodia and subsequently counterstained with DAPI (blue) and photographed (magnification x1,000). AZA197 leads to changes in cellular morphology. Click here for file

Additional file 4: Figure S4 Analysis of AZA197-signal transduction effectors in HT-29 colon cancer cells. **A** Cdc42 levels were not changed in HT-29 cells treated with AZA197 compared to untreated cells. Means of 3 independent experiments are shown. **B**, **C** Analysis of PAK1 (B) and ERK (C) phosphorylation in HT-29 colon cancer cells after AZA197 treatment. Representative Western blot images and quantification of immunoblots stained with PAK1, phospho-PAK1/2 (pPAK), ERK and phospho-ERK (pERK) antibodies before and after treatment with 2, 5 and 10 μM AZA197 for 24 h. Cdc42 blockade reduces PAK1 and ERK phosphorylation in HT-29 cells (mean of 3 independent experiments) without affecting total protein levels. *, significantly different from control. **D** Analysis of CyclinD1 expression in HT-29 colon cancer cells following AZA197 treatment. Representative Western blot images and quantification of immunoblots stained with Cyclin D1 antibody before and after treatment with 2, 5 and 10 μM AZA197 for 24 h. Cyclin D1 levels were reduced following AZA197 treatment of HT-29 cells (mean of three independent experiments). *, significantly different from control. SP, specific protein; LC, loading control. Click here for file
