# Supplementary material for: Targeting Cdc42 with the small molecule drug AZA197 suppresses primary colon cancer growth and prolongs survival in a preclinical mouse xenograft model by downregulation of PAK1 activity
Source: J Transl Med. 2013 Nov 27;11:295. doi: 10.1186/1479-5876-11-295 (PMC4222769; doi:10.1186/1479-5876-11-295)
Supplement: Additional file 1: Figure S1 — Compound AZA197 promotes LDH release and inhibits Cdc42 activation. A Cytotoxicity was assessed by LDH release in HT-29 colon cancer cells after 24 h exposure to AZA197 (1–100 μM). Co, untreated control; DMSO, solvent control; *, significantly different from untreated control. B Rac1, Cdc42 and RhoA activation in HT-29 colon cancer cells after incubation (24 h) with different concentrations of compound AZA197. AZA197 suppresses Cdc42 activity in colon cancer cells in a dose-dependent manner. Means of three independent experiments are shown. *, significantly different from untreated control. [file 1479-5876-11-295-S1.pdf]

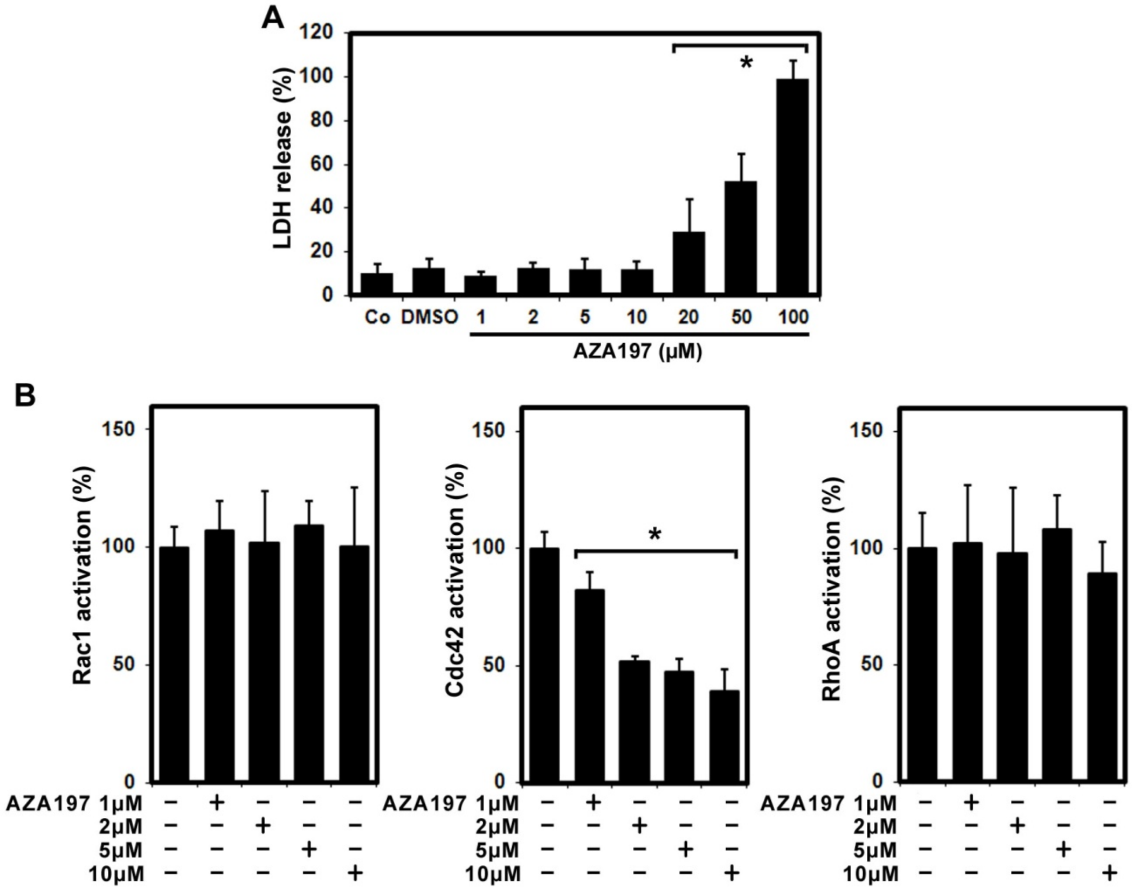

Additional Figure 1

Additional Figure 1 Compound AZA197 promotes LDH release and inhibits Cdc42 activation. A Cytotoxicity was assessed by LDH release in HT-29 colon cancer cells after 24 h exposure to AZA197 (1–100 µM). Co, untreated control; DMSO, solvent control; *, significantly different from untreated control. **B** Rac1, Cdc42 and RhoA activation in HT-29 colon cancer cells after incubation (24 h) with different concentrations of compound AZA197. AZA197 suppresses Cdc42 activity in colon cancer cells in a dose-dependent manner. Means of three independent experiments are shown. *, significantly different from untreated control.
