# Supplementary material for: Targeting Cdc42 with the small molecule drug AZA197 suppresses primary colon cancer growth and prolongs survival in a preclinical mouse xenograft model by downregulation of PAK1 activity
Source: J Transl Med. 2013 Nov 27;11:295. doi: 10.1186/1479-5876-11-295 (PMC4222769; doi:10.1186/1479-5876-11-295)
Supplement: Additional file 2: Figure S2 — Effects of Cdc42 inhibition by AZA197 on cell proliferation in HT-29 colon cancer cells. A Relative density of cancer cells up to 72 h following treatment with 1, 2, 5 and 10 μM AZA197 was measured using the WST-1 cell proliferation assay. AZA197 suppresses HT-29 colon cancer cell proliferation in a dose-dependent manner. Means of three independent experiments are shown. *, significantly different from control. [file 1479-5876-11-295-S2.pdf]

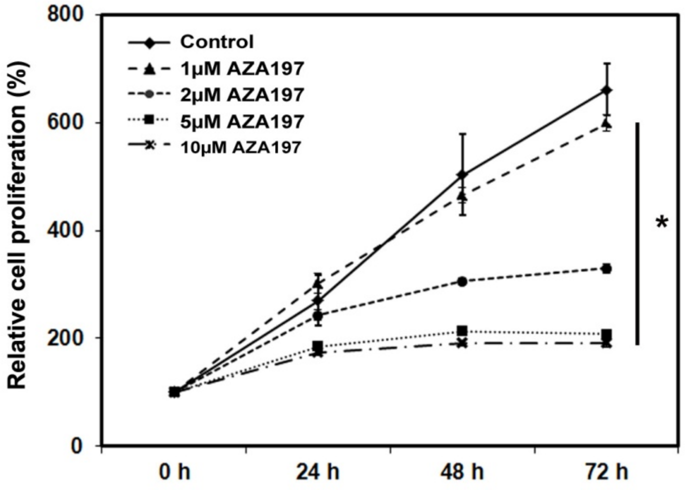

Additional Figure 2

Additional Figure 2 Effects of Cdc42 inhibition by AZA197 on cell proliferation in HT-29 colon cancer cells. A Relative density of cancer cells up to 72 h following treatment with 1, 2, 5 and 10 µM AZA197 was measured using the WST-1 cell proliferation assay. AZA197 suppresses HT-29 colon cancer cell proliferation in a dose-dependent manner. Means of three independent experiments are shown. *, significantly different from control.
