# Supplementary material for: Targeting Cdc42 with the small molecule drug AZA197 suppresses primary colon cancer growth and prolongs survival in a preclinical mouse xenograft model by downregulation of PAK1 activity
Source: J Transl Med. 2013 Nov 27;11:295. doi: 10.1186/1479-5876-11-295 (PMC4222769; doi:10.1186/1479-5876-11-295)
Supplement: Additional file 3: Figure S3 — Cdc42 blockade reduces colon cancer cell migration, invasion and affects actin cytoskeleton reorganization. A Representative images of migrated HT-29 colon cancer cells from an in vitro migration assay are shown. Colon cancer cells were treated with 1, 2 or 5 μM AZA197 for 24 h and migrated cancer cells quantified subsequently by in vitro migration assays. Data were collected from five individual consecutive fields of view (40x) from three replicate Boyden chambers. *, significantly different from control. B The invasive capacity of HT-29 cells was determined in matrigel invasion assays. Invaded HT-29 cells were quantified from five individual consecutive fields of view (100x) from three replicate chambers. *, significantly different from control. C Effect of AZA197 treatment on cell morphology, filopodia formation and actin reorganization. HT-29 colon cancer cells were plated on fibronectin/gelatin coated cell culture chambers and incubated with 2, 5 and 10 μM AZA197 for 24 h. Paraformaldehyde fixed cells were stained with Atto-488 phalloidin (F-actin, green) to visualize the polymerized actin cytoskeleton and filopodia and subsequently counterstained with DAPI (blue) and photographed (magnification x1,000). AZA197 leads to changes in cellular morphology. [file 1479-5876-11-295-S3.pdf]

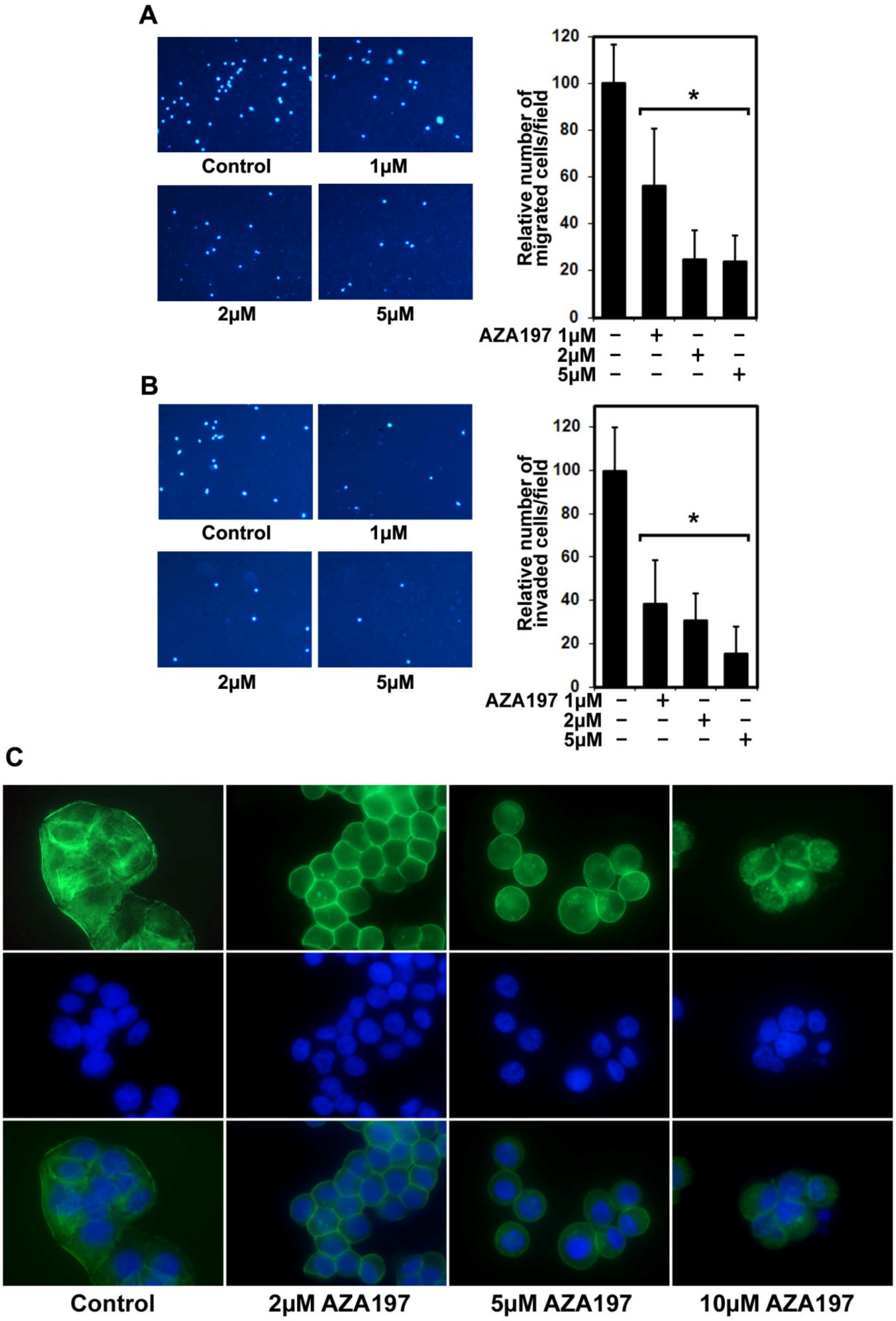

Additional Figure 3

Additional Figure 3 Cdc42 blockade reduces colon cancer cell migration, invasion and affects actin cytoskeleton reorganization. A Representative images of migrated HT-29 colon cancer cells from an *in vitro* migration assay are shown. Colon cancer cells were treated with 1, 2 or 5 µM AZA197 for 24 h and migrated cancer cells quantified subsequently by *in vitro* migration assays. Data were collected from five individual consecutive fields of view (40x) from three replicate Boyden chambers. *, significantly different from control. **B** The invasive capacity of HT-29 cells was determined in matrigel invasion assays. Invaded HT-29 cells were quantified from five individual consecutive fields of view (100x) from three replicate chambers. *, significantly different from control. **C** Effect of AZA197 treatment on cell morphology, filopodia formation and actin reorganization. HT-29 colon cancer cells were plated on fibronectin/gelatin coated cell culture chambers and incubated with 2, 5 and 10 µM AZA197 for 24 h. Paraformaldehyde fixed cells were stained with Atto-488 phalloidin (F-actin, green) to visualize the polymerized actin cytoskeleton and filopodia and subsequently counterstained with DAPI (blue) and photographed (magnification x1,000). AZA197 leads to changes in cellular morphology.
