# Supplementary material for: Targeting Cdc42 with the small molecule drug AZA197 suppresses primary colon cancer growth and prolongs survival in a preclinical mouse xenograft model by downregulation of PAK1 activity
Source: J Transl Med. 2013 Nov 27;11:295. doi: 10.1186/1479-5876-11-295 (PMC4222769; doi:10.1186/1479-5876-11-295)
Supplement: Additional file 4: Figure S4 — Analysis of AZA197-signal transduction effectors in HT-29 colon cancer cells. A Cdc42 levels were not changed in HT-29 cells treated with AZA197 compared to untreated cells. Means of 3 independent experiments are shown. B, C Analysis of PAK1 (B) and ERK (C) phosphorylation in HT-29 colon cancer cells after AZA197 treatment. Representative Western blot images and quantification of immunoblots stained with PAK1, phospho-PAK1/2 (pPAK), ERK and phospho-ERK (pERK) antibodies before and after treatment with 2, 5 and 10 μM AZA197 for 24 h. Cdc42 blockade reduces PAK1 and ERK phosphorylation in HT-29 cells (mean of 3 independent experiments) without affecting total protein levels. *, significantly different from control. D Analysis of CyclinD1 expression in HT-29 colon cancer cells following AZA197 treatment. Representative Western blot images and quantification of immunoblots stained with Cyclin D1 antibody before and after treatment with 2, 5 and 10 μM AZA197 for 24 h. Cyclin D1 levels were reduced following AZA197 treatment of HT-29 cells (mean of three independent experiments). *, significantly different from control. SP, specific protein; LC, loading control. [file 1479-5876-11-295-S4.pdf]

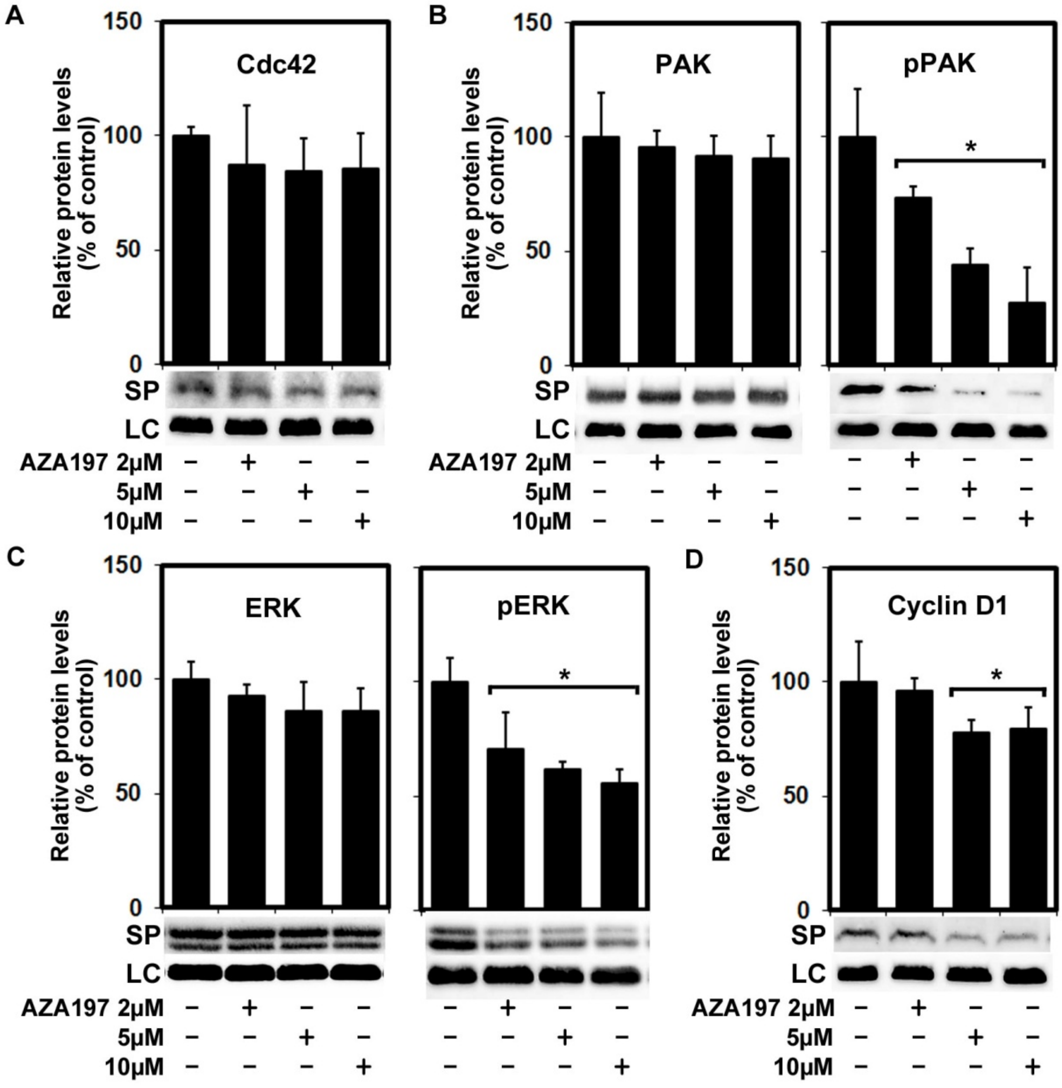

Additional Figure 4

Additional Figure 4 Analysis of AZA197-signal transduction effectors in HT-29 colon cancer cells. A Cdc42 levels were not changed in HT-29 cells treated with AZA197 compared to untreated cells. Means of 3 independent experiments are shown. **B, C** Analysis of PAK1 (B) and ERK (C) phosphorylation in HT-29 colon cancer cells after AZA197 treatment. Representative Western blot images and quantification of immunoblots stained with PAK1, phospho-PAK1/2 (pPAK), ERK and phospho-ERK (pERK) antibodies before and after treatment with 2, 5 and 10 μM AZA197 for 24 h. Cdc42 blockade reduces PAK1 and ERK phosphorylation in HT-29 cells (mean of 3 independent experiments) without affecting total protein levels. *, significantly different from control. **D** Analysis of CyclinD1

expression in HT-29 colon cancer cells following AZA197 treatment. Representative Western blot images and quantification of immunoblots stained with Cyclin D1 antibody before and after treatment with 2, 5 and 10 µM AZA197 for 24 h. Cyclin D1 levels were reduced following AZA197 treatment of HT-29 cells (mean of three independent experiments). *, significantly different from control. SP, specific protein; LC, loading control.
